# Evaluation of Phage Therapy in the Context of *Enterococcus faecalis* and Its Associated Diseases

**DOI:** 10.3390/v11040366

**Published:** 2019-04-20

**Authors:** Andrei S. Bolocan, Aditya Upadrasta, Pedro H. de Almeida Bettio, Adam G. Clooney, Lorraine A. Draper, R. Paul Ross, Colin Hill

**Affiliations:** 1 APC Microbiome Ireland, University College Cork, Cork T12 YT20, Ireland; andrei.s.bolocan@gmail.com (A.S.B.); aupadrasta@gmail.com (A.U.); pedro.almeida.bettio@gmail.com (P.H.d.A.B.); adam.clooney@ucc.ie (A.G.C.); L.Draper@ucc.ie (L.A.D.); p.ross@ucc.ie (R.P.R.); 2 School of Microbiology, University College Cork, Cork T12 YN60, Ireland; 3 Teagasc Food Research Centre, Moorepark, Fermoy, Cork P61 C996, Ireland

**Keywords:** phage therapy, *E. faecalis*, OrthoMCL

## Abstract

Bacteriophages (phages) or bacterial viruses have been proposed as natural antimicrobial agents to fight against antibiotic-resistant bacteria associated with human infections. *Enterococcus faecalis* is a gut commensal, which is occasionally found in the mouth and vaginal tract, and does not usually cause clinical problems. However, it can spread to other areas of the body and cause life-threatening infections, such as septicemia, endocarditis, or meningitis, in immunocompromised hosts. Although *E. faecalis* phage cocktails are not commercially available within the EU or USA, there is an accumulated evidence from in vitro and in vivo studies that have shown phage efficacy, which supports the idea of applying phage therapy to overcome infections associated with *E. faecalis*. In this review, we discuss the potency of bacteriophages in controlling *E. faecalis*, in both in vitro and in vivo scenarios. *E. faecalis* associated bacteriophages were compared at the genome level and an attempt was made to categorize phages with respect to their suitability for therapeutic application, using orthocluster analysis. In addition, *E. faecalis* phages have been examined for the presence of antibiotic-resistant genes, to ensure their safe use in clinical conditions. Finally, the domain architecture of *E. faecalis* phage-encoded endolysins are discussed.

## 1. Introduction

*Enterococcus* is a genus of gram-positive non-spore-forming bacteria that typically inhabit the gastrointestinal tract (GIT), which currently contains thirty five well-recognized species [1], including *Enterococcus faecalis*. The enterococci possess a remarkable ability to adapt to different environments and have a propensity to acquire antibiotic resistance, which has led to the emergence of multi-drug resistant variants, across the genus [1]. *E. faecalis* is mainly described as a core commensal member of the human gut, but it can also act as an opportunistic pathogen and translocate across the mucosal barrier to cause systemic infections [2,3]. More than 90% of the bacterial isolates frequently recovered from clinical specimens (blood, and other infectious site samples) are *E. faecalis* and *E. faecium* [4,5]. Life-threatening infections generally linked to *E. faecalis* include endocarditis, bacteremia, urinary tract infections, meningitis, and root canal infections. In contrast, *E. faecalis* Symbioflor 1 strain (Symbiopharm, Herborn, Germany) has been demonstrated to be a safe and effective probiotic and a few other enterococcal strains have been used as starter cultures in the cheese industry [6]. However, the genus *Enterococcus* is not listed in the Qualified Presumption of Safety (QPS) of the European Food Safety Authority, nor does it have a generally regarded as safe (GRAS) status [6]. Hence the continued use of enterococci in traditional fermented foods and as probiotics, is controversial, because of their association with human infections [7].

Antimicrobial resistance (AMR) causes 700,000 global deaths each year, and it is estimated that it will rise to 10 million deaths by 2050 [7,8]. The high prevalence of Multi-Drug Resistant (MDR) bacteria and inefficiency of available antibiotics to overcome infectious diseases, has inspired a search for viable alternatives. Bacteriophages, also known as phages, and their associated cell wall lysing enzymes (endolysins), have the potential to be useful tools to combat MDR pathogens [9,10,11].

Phages are prokaryotic viruses that have the ability to infect and replicate within their host bacterial cell, and to subsequently lyse the cell, to release their progeny. Based on their replication strategy, phages can undergo two different life cycles; the lytic (virulent) and the lysogenic (temperate). Naturally virulent phages are suitable candidates for phage therapy, but temperate phages are not as useful. However, genome engineering strategies can be applied to convert temperate phages to virulent, for their effective use in phage therapy [12]. Phage therapy is described as the application of phages to treat bacterial infections [13,14]. There are some indications that phages could be suitable alternatives to combat *Enterococcus*-associated infections [2,15,16,17,18]. In this review, we focus on (i) phage therapy to treat *E. faecalis* infections using in vitro and in vivo models; (ii) the genetic relationships between currently isolated *E. faecalis* bacteriophages; (iii) identification of candidates suitable for phage therapy; (iv) *E. faecalis* phages endolysins as alternative to phage therapy; and (v) conclusions and recommendations for further development of *E. faecalis* phage therapy.

## 2. The Necessity of *E. faecalis* Phage Therapy

*E. faecalis* is one of the first colonizers of the human GIT and it plays a role in intestinal immune development at the very early stages of life [19]. *E. faecalis* is a ubiquitous microorganism that possesses the ability to survive and persist in a broad range of environments. In susceptible hosts, *E. faecalis* can act as an opportunistic pathogen, causing severe infections, including urinary tract infections (UTIs), endocarditis, bacteremia, catheter-related infections, wound infections, and intra-abdominal and pelvic infections [1].

An important question is, what makes this bacterium an opportunistic pathogen and under what circumstances? The key factors linked to the pathogenic role of *E. faecalis* in the GIT is its ability to generate reactive oxygen species (ROS) and extracellular superoxide, which can cause genomic instability and damage to the colonic DNA [20]. Opportunistic infection has been associated with the production of virulence factors, adherence to Caco-2 and HEP-2 cells, capacity for biofilm formation and resistance to antimicrobials [21,22,23]. Numerous virulence factors have been identified that are associated with a wide range of *E. faecalis* infections; namely, aggregation substance (AS), adhesion to collagen of *E. faecalis* (Ace), cell wall glycopeptides, gelatinase (GelE) and biofilm-associated Pili (Ebp), Enterococcal fibronectin-binding protein A (EfbA), membrane metalloprotease (Eep), and biofilm formation. AS is a pheromone-inducible plasmid-encoded cell surface protein, involved in bacterial aggregation during conjugation, via binding to the enterococcal binding substance (EBS) [22,23,24,25,26]. There are three AS proteins (Asa1, Asc10, and Asp1), which belong to a family of surface adhesions and are highly similar to each other. These factors are responsible for the initial adherence and biofilm formation at infected sites [25,27]. Other important cell wall-associated virulence factors are pili and fimbriae, which are anchored to the outer cell surface of the bacterium and aid the bacterium to adhere to host cells. In *E. faecalis*, these are encoded by a three-gene locus (ebpABC), with an associated enzyme sortase, srtC. This ebpABC locus has also been shown to encode proteins involved in biofilm formation [24,28].

Other virulence factors such as Ace, a cell-wall anchored adhesion, plays a pivotal role in in vitro adherence [27,29]. Similarly, EfbA, located on the outer cell membrane, confers adhesion to the host glycoprotein fibronectin [30]. One more critical virulence factor is GelE, an extracellular zinc-metallo protease that contributes to the degradation of various host proteins, such as collagen, fibrinogen, fibrin, and immune complement components C3 and C3a. Many of these factors associated with virulence are also known to promote biofilm formation in *E. faecalis*, suggesting that biofilms are crucial to development of severe infections [31].

In addition, *E. faecalis* is intrinsically resistant to numerous antibiotics, such as penicillin, ampicillin, piperacillin, imipenem, and vancomycin—which have only bacteriostatic rather than bactericidal effects [32]. Over the last decade vancomycin-resistant *E. faecalis* (VREF), together with the other vancomycin-resistant enterococci (VRE), have generated much concern. In the context of a cumulative mortality rate of 20–40% for infective endocarditis, generated by *E. faecalis* and *E. faecium*, *E. faecalis* accounts for approximately 97% of cases [33]. In contrast to that, in leukemia patients, the VR *E. faecium* is more prevalent, accounting for 84%, followed by *E. faecalis* accounting for 6% and the rest 10% was occupied by all other *Enterococcus* sp. [34] and these percentages slightly varied in different studies [35]. In addition, it has been reported that VR *E. faecium* was the leading cause of early infection-related mortality in older (≥60 years) acute leukemia patients, who were receiving induction chemotherapy [36]. Moreover, enterococcal bloodstream infections occurs frequently in patients with acute leukemia, and causes significant morbidity and mortality (87% due to *E. faecium*, while only 13% due to *E. faecalis*) [37]. However, the role of *E. faecalis* and *E. faecium* in colorectal cancer and other diseases such as inflammatory bowel disease (IBD), remains unclear, and their involvement in colorectal cancer is still under investigation [38]. It is presumed that it is the inefficient activity of β-lactams, as well as the biofilm-forming ability of *E. faecalis* which makes these infections difficult to treat. Often, combinations of antibiotic therapies are required for treatment of severe infections associated with *E. faecalis*. However, even these antibiotic treatment options are limited, considering that 50% of isolates exhibit a high-level of aminoglycoside resistance, mediated by aminoglycoside-modifying enzymes, which eliminate the synergistic bactericidal effect, usually seen when a cell wall-active agent is combined with an aminoglycoside [33,39].

## 3. Strategies for Obtaining *E. faecalis* Phages for Phage Therapy

There are several advantages associated with bacteriophages over antibiotics to treat bacterial infections. For example, unlike antibiotics, bacteriophages are highly specific to their corresponding target and, thus, do not perturb indigenous microbial communities [13,40,41,42]. Phages targeting *Enterococcus* spp. have been isolated from various sources, like sewage, animal yard effluents, human feces, urogenital secretions or by inducing chromosomally integrated prophages [17,43,44,45,46].

In general, plaque and spot assays are the methods applied by researchers to isolate phages, using bacterial hosts of interest. In an attempt to increase the recovery of phages from environments where they are scarce, a pre-enrichment step has been widely used, prior to plaque/spot assay. In the case of *E. faecalis*, typically, vancomycin-resistant strains or other clinical isolates have been used for screening, in order to realize the potential of phages as novel therapeutics [38,41].

Many factors can affect the process of phage isolation. For example, poor or invisible plaque morphology, difficulty in obtaining confluency of bacterial lawns, poor enrichment of samples containing very low numbers of phages, or sample availability [47]. Furthermore, bacterial host strains might adapt to routine laboratory culturing practices resulting in changes to their cell physiology. Such genotypic and phenotypic changes which occur during sub-culturing, can reduce the chances for the discovery new phages. To overcome such hurdles, Purnell et al. [37], suggest the isolation of target bacterial hosts, and their cognate bacteriophages, from the same sample, to achieve a higher success rate. Therefore, it is advisable to obtain a fresh culture from the glycerol stock and avoid multiple sub-culturing and serial broth-to-broth transfers, prior to phage isolation. In addition, bacteria can rapidly evolve to overcome phage infection by means of spontaneous mutation, or by acquiring CRISPR-*cas* mediated adaptive immunity, resulting in bacteriophage-insensitive mutants (BIMs) [48,49,50]. In addition, since multiple bacterial strains can be involved in diseases, the application of phage cocktails are deemed to be more appropriate over single-phage preparations, in therapeutic interventions [16].

## 4. Orthocluster Analysis of *E. faecalis* Phages

On the 30 December 2018, fifty-four *Enterococcus* phage genome sequences were available (http://millardlab.org/bioinformatics/bacteriophage-genomes/), of which 89% had *E. faecalis* and 11% had *E. faecium* as a target (Table S1). Usually, these phages infect both species at varying efficiencies [16,17,51,52,53,54].

**Figure 1:**
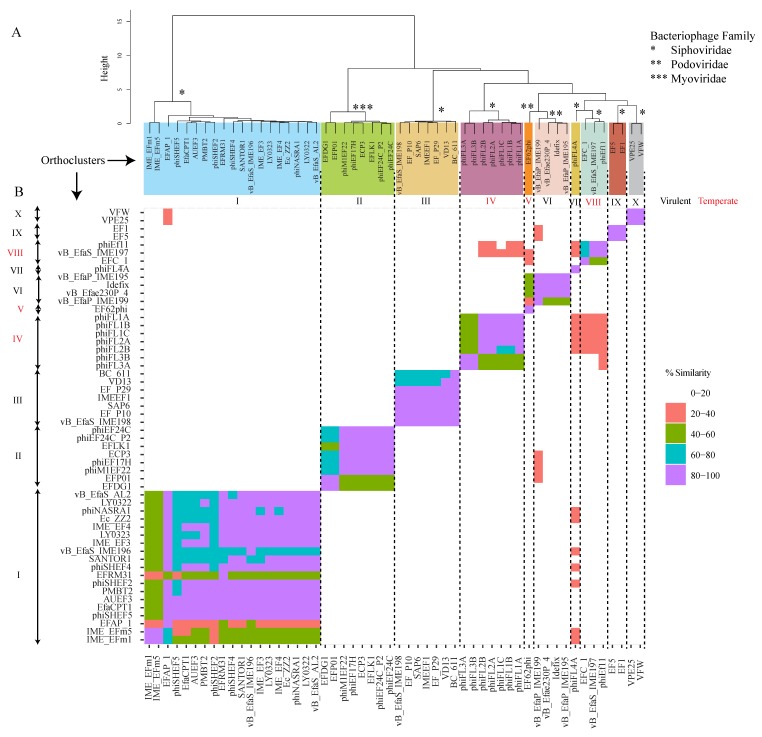
Genomic comparison of *Enterococcus* phages. (**A**) Neighbor-joining tree based on the percentage of shared orthologous genes (1000 bootstrap replicates); squares indicate the 10 phage putative orthoclusters. (**B**) Dot plot comparison of amino acids identity among the 10 orthoclusters; genes that share more than 40% homology were considered as being part of the same orthologous group. The vertical axis shows phage clusters and phage IDs.

To determine the gene content relationship between these bacteriophages, a cluster analysis was performed on the basis of the percentage of shared orthologous genes. For the orthocluster analysis, the phage genomes were downloaded from the NCBI database, and potential Open Reading Frames (ORFs) were predicted by Prodigal [55]. Identification of the bacteriophage protein Orthologous Groups (OG, cluster of proteins from at least two phages) was performed, using orthoMCL [56]. OrthoMCL phage clusters identified from this analysis were defined as “orthoclusters”. This analysis allowed the identification of ten distinct and well-supported (100% bootstrap support) clusters of *Enterococcus* phage genomes. Of the fifty-four *Enterococcus* phage genomes, fifty-two fell into one of the ten distinct clusters, designated as orthoclusters I–V, VII, IX–X, as depicted in Figure 1. The remaining two phages used in this analysis, did not cluster with any other phages. Therefore, we hypothesize that the phages EF62phi and phiFL4, formed two different orthoclusters, V and VII, respectively. The distinct orthoclusters, typically contain phages of the same family, with similar genome size, GC content and morphology. The clustering was in good agreement with classical taxonomical phage families, as determined by the morphology and genome analysis—virulent *Myoviridae* family—orthocluster II, virulent *Siphoviridae* family—orthoclusters I, III, V, VII, IX, and X, temperate *Siphoviridae* family—orthoclusters IV and VIII, and temperate *Podoviridae* family—orthocluster V, and virulent *Podoviridae* family—orthocluster VI.

With respect to phage therapy, orthoclusters comprising native virulent phages, are of immense interest. Of the *Enterococcus* phages characterized to date, 77% are known to be virulent, and belong to the orthoclusters I, II, III, IV, VI, IX, and X. Although temperate phages have less obvious usefulness with respect to phage therapy, molecular mechanisms of phage conversion from temperate to virulent, might make this possible. 

**Figure 2:**
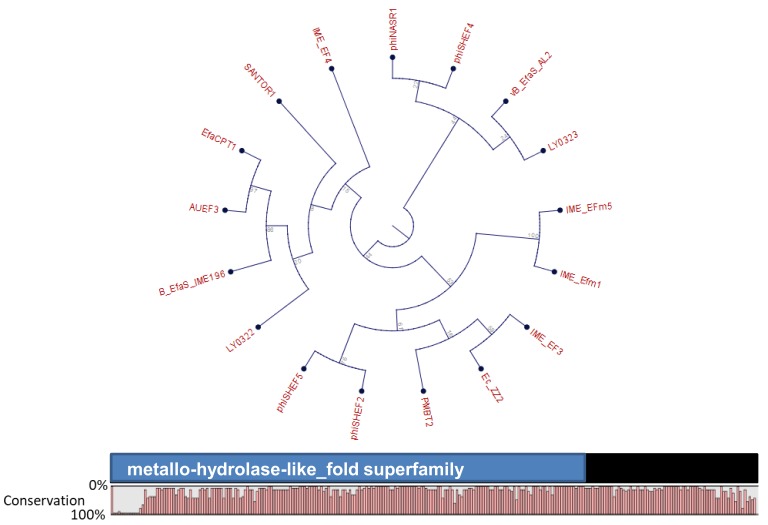
Maximum likelihood phylogenetic analysis sequence relatedness of the *Enterococcus faecalis* phage putative metallo-beta-lactamase gene (orthologous group 32); tree node labels represent bootstrap values.

Orthocluster I, which is supported by a bootstrap value of 1000, contains 19 phages belonging to the *Siphoviridae* family. This orthocluster is particularly interesting as the phages differ significantly from each other, in terms of their genome length and mean GC content, features which are conserved among the other orthoclusters. The genome sizes range from ~17 kb to ~42 kb, and the mean GC content varies from 17.35% to 36.7%. The suitability of these phages for phage therapy is questionable, as the orthologous group 32, which belongs to orthocluster I, contains the putative metallo-beta-lactamase gene, a gene related to antibiotic resistance (Figure 2) [57,58]. All phages harbor this gene, except for EFRM31 and EFAP_1, within the orthocluster I. However, the functionality of this gene is currently unknown. Further studies are warranted to evaluate these phages and their involvement in antibiotic gene dissemination in the gut. In addition, gene editing tools could be applied to either delete or inactivate the metallo-beta-lactamase gene, before considering therapeutic applications. A study by Nezhad Fard et al. [59], demonstrated that the phage EFRM31 was efficient at transducing gentamicin resistance to multiple enterococcal species. In fact, this was the first example of inter-species host range generalized transduction, and thus, it did not support a role for such phages in therapeutic applications.

Interestingly, Orthocluster II incorporates all the *Myoviridae* phages described so far, which infect *E. faecalis*. These phages can infect and proliferate in multiple strains of *E. faecalis* and *E. faecium* strains. The size of the genomes ranged between ~130 kb to ~150 kb, and the mean GC content was estimated to be 35.3% to 37.2%. These phages were related to SPO1-like viruses, such as the *Staphylococcus* phage K, *Listeria* phage P100, and *Lactobacillus* phage LP65. Interestingly, no *E. faecalis* temperate phages belonging to the *Myoviridae* family have ever been described [16,60].

Orthocluster III contains the most studied *E. faecalis* virulent phages from the *Siphoviridae* family (genus *Sap6virus*). The size of the genomes ranged between ~53 kb to ~59 kb, and the mean GC content was estimated to be 39% to 40%. These phages exhibited a broad host range and a high level of efficiency in in vitro and in vivo studies, which have been discussed in more detail, later on. Genome analysis did not reveal any putative virulence factors or antibiotic-resistant genes, and to date no transduction potential has been described. Members of this orthocluster should, therefore, be considered and studied with respect to their therapeutic potential [61,62]. 

The phages from Orthocluster IV were induced using norfloxacin and UV from bacteremia isolates of the *Enterococcus* sp. These temperate phages belonged to the *Siphoviridae* family, with a genome size of 30–40 kb, and a mean GC content of 30%–40%. Currently, only virulent phages have been considered as suitable candidates for phage therapy, but there is a possibility to convert these lysogenic phages to virulent entities, which would allow us to investigate these phages in the context of phage therapy. However, the use of genetically-modified phages, is not acceptable, for now [12]. Further inspection of the orthocluster IV harboring temperate phages, revealed their ability to pack its bacterial host DNA, a generalized transduction potential event observed in some other temperate phages, as well. As a result, these phages are not suitable for phage therapy. It is unfortunate that on rare occasions generalized transduction events have also been observed in some virulent phages [43]. 

The *Podoviridae* phage, EF62phi (~30 kb, mean GC content 32.7%) which forms the putative orthocluster V, is a pseudotemperate linear bacteriophage identified in the genome of *E. faecalis* strain 62, isolated from a healthy Norwegian infant. EF62ph is the only pseudotemperate enterococcal phage described to date. EF62ph is maintained in the bacterial genome by means of RepB and a toxin–antitoxin system [63]. There have been no studies, so far, on pseudotemperate enterococcal phages and their involvement in phage therapy.

Orthocluster VI is comprised of the *Podoviridae* phages, of the genus *Ahjdlikevirus*. These phages have been isolated from sewage, and infect both *E. faecalis* and *E. faecium* strains. The size of the genomes range from ~17 kb to ~18 kb, and have a mean GC content of 33.2% to 34.6%. With no evidence of antibiotic-resistance-associated genes or transduction potential, these phages should be explored further for potential therapeutic applications [17,54,64].

The phage phiFL4A, which forms the putative orthocluster VII (*Siphoviridae* family, *Phifelvirus* genus, 37 kb, mean GC content 37.8%) was induced from bacteremia isolates, using mitomycin C, in the same study as that of the phages of orthocluster IV. This phage is also temperate and has the ability of generalized transduction and, therefore, is not eligible for phage therapy [43].

Orhocluster VIII contains three temperate prophages and is part of the *Siphoviridae* family. phiEf11 was induced with mitomycin C from the root isolate *E. faecalis* TUSoD11 [65], EFC1 was induced with mitomycin C from the raw milk isolate *E. faecalis* KBL101 [66] and vB_EfaS_IME197 was isolated from sewage. The size of the genomes range from ~40 kb to ~42 kb and the mean GC content from 34% to 35%. This group is particularly interesting from the point of view of phage therapy, as Ef11 phage have been converted from temperate to virulent, followed by successful testing against *E. faecalis*. Therefore, the temperate phages from this orthocluster opens the direction for a new type of *E. faecalis* phage therapy, based on genetically engineered phages [67,68,69] 

The phages that form orthocluster IX, EF1, and EF5, were previously annotated as part of the *Myoviridae* family. However, our genome annotation using RASTtk and BLAST suggest that these two virulent phages are part of the *Siphoviridae* family. By comparison with the other *Siphoviridae* virulent *E. faecalis* phages, these two phages have a large genome of 141.996 kb, with a mean content GC of 31.9%. Larger genomes are typical for *Myoviridae* family, which may be the reason for their previous attribution in the database. No therapeutic studies have been performed using these phages and, therefore, their potential role in phage therapy could not be predicted. Despite this, our genome analysis did not reveal any genes that would hinder further research of these phages for therapeutic potential.

Phages VPE25 and VFW formed orthocluster X. They were isolated from sewage and shared 95% homology at the nucleotide level. The size of the genomes of both phages was ~86 kb, with a mean GC of 33.2%. VPE25 and VFW were obligate lytic and their isolation, using VR *E. faecalis* V583 as a host, suggested them to be putative candidates for therapy [70]. 

Phages from each of the described orthocluster are now discussed in more details, with respect to the published in vitro and in vivo phage therapy studies.

## 5. *E. faecalis* Phage Therapy in In Vitro Models

### 5.1. Biofilm Eradication

Various studies describe the ability of single phage or phage cocktails in the treatment of bacterial biofilms. For example, biofilms formed by pathogenic bacteria *Streptococcus mutants* [71], *E. coli* [72], *Pseudomonas aeruginosa* [73], *Staphylococcus aureus* [74], and *E. faecalis* [75], can be disrupted by phages. Phage treatment is more efficient against biofilms, compared to conventional antibiotics, since, as the phages infect the bacteria from the upper layer, upon replication they release a new virion progeny, which subsequently attacks the bottom layer(s). As a result of this layer-by-layer mode of action, the biofilms are effectively eradicated [75,76]. Microtiter plates are the most commonly used method for studying biofilm formation, and to test the activity of antimicrobial compounds. More advanced techniques like confocal microscopy can also be applied for the visualization of biofilm matrices, before and after phage treatment [77]. Using this method, the efficiency of phage EFDG1 (orthocluster II) to reduce two-week-old biofilms of *E. faecalis* V583 has been described [18]. The genetically-engineered orthocluster VIII phage phiEf11 (phiEf11/phiFL1C(Δ36)PnisA [67]), reduced the static biofilm of *E. faecalis* strains JH2-2 (pMSP3535 nisR/K) and V583 (pMSP3535nisR/K), which had formed on coverslips. After 24 and 48 h of incubation, a 10–100-fold decrease in viable cells (CFU/biofilm) was observed [69].

### 5.2. Human Root Canal Model (In Vitro/Ex Vivo)

*E. faecalis* has been found, over time, to be more prevalent (24% to 77% of cases) in asymptomatic and persistent endodontic infections [78,79]. The extreme survival ability and highly adaptive nature of *E. faecalis* in harsh environments, allows the bacterium to cause persistent infections in root canals. Furthermore, it can resist nutritional deprivation and invade dental tubules to form endodontic biofilms. In this scenario, treatment with 2% chlorhexidine, combined with sodium hypochlorite, is generally effective. However, a number of failures have been recorded in endodontic treatment, due to technical difficulties associated with dental practices [78,80]. Therefore, the development of alternative strategies are necessary to prevent such situations. In this regard, the efficacy of phage treatment has been evaluated using an ex vivo two chamber bacterial leakage model of human teeth [18]. No turbidity was observed in the obturated root canals, which were subjected to 10^8^ PFU/mL of EFDG1 phage (orthocluster II) irrigation and the results also indicated a 7-log reduction of bacterial leakage, from the root apex, when compared to the control. In a similar study, Paisano et al. [81] showed that a phage lysate of 2 × 10^8^ PFU/mL was able to significantly inhibit *E. faecalis* in human dental roots inoculated for 6 days with a suspension of *E. faecalis* ATCC 29212 at the three different multiplicities of infection; 0.1, 1.0, and 10.0. Moreover, in the study of Tinoco et al [12]. extracted human dentin root segments were cemented into a sealable double-chamber and inoculated for 7 days, with an overnight suspension of either VR *E. faecalis* V583, or *E. faecalis* JH2-2, which is vancomycin sensitive, but resistant to fusidic acid and rifampin. The treatment with genetically-engineered phage, phiEf11/phiFL1C (Δ36)PnisA, generated a reduction of 18% for the JH2-2-infected models, and by 99% for the V583-infected models. These examples certainly strengthen the efficacy of phage therapy in the treatment of *E. faecalis* root canal infections.

### 5.3. Fibrin Clot Model

Clots are gel-like clumps of blood that occurs when thrombin converts fibrinogen to fibrin, a structural protein that assembles into a polymer [82]. An in vitro fibrin clot model has been successfully used to test the role of antibiotics in the treatment of bacterial endocarditis [83], demonstrating the in vitro clotting ability of bacterial strains *Bacillus cereus* [84], *Staphylococcus aureus* [85], *E. faecalis* [86], and *E. faecium* [83,84]. Recently, the in vitro fibrin clot model has been used to demonstrate the efficacy of individual phages and phage cocktails [16]. The authors spiked the plasma with vancomycin-resistant and sensitive *E. faecalis* strains, and triggered the plasma coagulation with the addition of bovine thrombin and CaCl_2_. The resultant clots were subjected to a 10^8^ PFU/mL bacteriophage treatment. Bacterial counts were significantly reduced by 3–6 logs, after treatment with phage(s) EFDG1 and EFLK1 (orthocluster II).

### 5.4. E. faecalis Phages as Biocontrol Agents

Bacteriophages have long been recognized as effective biological entities in the control of undesired foodborne bacteria. In 2007, a *Listeria*-specific bacteriophage preparation, Listex P100, obtained U.S. FDA approval for use as a biopreservative, in ready-to-eat meat products (U.S. Food and Drug Administration, 2007). In a recent study, phage Q69 has been shown to be effective against *E. faecalis*, in a cheese model system. This phage significantly reduced *E. faecalis* numbers and subsequently eliminated the accumulation of toxic biogenic amine tyramine, during cheese ripening [87].

## 6. *E. faecalis* Phage Therapy in In Vivo Models

To date, we are only aware of a single human study describing the phage treatment *of E. faecalis* associated chronic prostatitis (Table 1). Three subjects were selected for phage therapy who had failed to respond to antibiotic, auto-vaccine, and laser bio-stimulation treatments. During phage treatment, 10 mL of bacterial phage lysate was rectally applied, twice daily, for 30 days. In all three cases, the pathogen was eradicated, clinical symptoms abated, and early disease recurrence was not observed [88]. 

Other positive results obtained on treating infectious disease unresponsive to antibiotics, caused by other bacteria, such as *S. aureus*, *E. coli*, *Klebsiella*, *Proteus*, *Pseudomonas*, and *Enterobacter*, support the idea of using phage therapy against antibiotic-resistant *E. faecalis* [93]. All highlight the efficiency of phages in disease resolution, and as future options for treating multi-drug-resistant bacterial infections. Another example describes a life-threatening multi-drug-resistant pathogen *Acinetobacter baumannii* infection, which was treated with an intravenous bacteriophage cocktail. This reversed the patient’s clinical trajectory, cleared the *A. baumannii* infection, and restored the individual from a state of coma to complete health [94]. More clinical scenarios like these will undoubtedly open new avenues for phages or phage-derived enzybiotics as biotherapeutics, to combat situations where antibiotic treatments are no longer viable.

### 6.1. Vertebrate Models

Meanwhile, some studies have shown the efficacy of phages, in vivo, against *E. faecalis*, using mouse models (Table 1). An intraperitoneal application of phages, significantly rescued mice, when deliberately challenged with the *E. faecalis* EF14 and *E. faecalis* VRE2 strains [95]. Similarly, another study has showed that mice treated with different phage doses were protected from the VREF systemic infection, and alleviated the gut microbial imbalance that occurred as a result of infection [91]. In another study, a single dose of the lytic phage cocktail was effective in completely reversing a 100% mortality in a septic peritonitis mouse model caused by VREF, and without causing any collateral damage to the gut microbiome [60]. Furthermore, phage therapy has proven to be safe and effective in treating *E. faecalis*-induced bacteremia [90] and sepsis [52], in mouse models.

### 6.2. Invertebrate Models

The larvae of wax moth *Galleria mellonella* has been used as a model system to examine pathogenesis of many bacteria, such as *S. aureus*, *P. aeruginosa*, *L. monocytogenes*, *Klebsiella pneumoniae*, *E. faecalis*, and *E. faecium*, and the fungi *Candida albicans* and *Aspergillus fumigatus* [96,97,98,99,100]. This model involves monitoring *G. mellonella* caterpillars infected with bacterial culture, followed by the administration of a test drug or saline solution as a negative control. A number of *E. faecalis* virulence gene factors have been associated with larval mortality [101]. This method has been demonstrated as a suitable model for studying *E. faecalis*-drug interaction, for example, studies have used distamycin, linezolid, rifampicin, and extracts of *Zingiber officinale* [101,102,103]. The most significant advantage of this model is that it allows a precise measurement of the inoculum and the quantity of the administrated drug, over time. Not only are promising results obtained using this larval model, but it involves simple methodological approaches. To date, there are no reports of phages treatment of *E. faecalis* in *G. mellonella*. However, Yasmin et al. [43] infected *G. mellonella* with *E. faecalis* JH2-2 lysogenized by phiFL3A and phiFL3B (orthocluster IV), and found that it increased the mortality of caterpillars. Conversely, some of the other lysogens obtained in the same study, but with different phages, such as phiFL1B and phiFL2B (orthocluster IV), and phiFL4A (putative orthocluster VII), did not show any death in the caterpillars, when compared to the JH2-2 generic strain group. This *G. mellonella* model could be a valuable tool to pre-screen the ability of phages in an in vivo scenario, before performing large scale animal trials. In fact, the *Galleria* larval model has been used to examine the therapeutic potential of bacteriophages against other bacterial pathogens, such as *C. difficile* [104], *Burkholderia cepacia* [105], *Pseudomonas aeruginosa* [106], *Escherichia coli*, *K. pneumoniae*, *Enterobacter cloacae* [100], and *Cronobacter sakazakii* [107].

## 7. *E. faecalis* Phage Endolysins as Viable Alternatives for Phage Therapy

Endolysins, also termed phage lysins, have the ability to degrade the peptidoglycan layer of bacterial cell walls, leading to cell death. These phage-derived enzymes allow the release of nascent virions, following intracellular replication [108]. Endolysins possess a wide degree of killing activity, which also makes them potential therapeutic agents. Considering the bottlenecks associated with the production and purification of phages, to ensure the removal of host-derived endotoxins for therapeutic use, endolysin manufacture is a less arduous process, with a potentially similar outcome. Moreover, with the advent of mass sequencing technologies and the availability of curated gene functional databases, it is now possible to access the genomes of uncultured phages and their enigmatic gene content, to develop potential lytic enzymes, without the necessity for phage isolation. In fact, an in silico examination of uncultured phage genomes, revealed enormous diversity among endolysins [109]. With a varied host specificity and domain architecture, the development of robust novel antimicrobials for future application are within our reach.

### 7.1. Domain Architecture of E. faecalis Phage Endolysins

**Figure 3:**
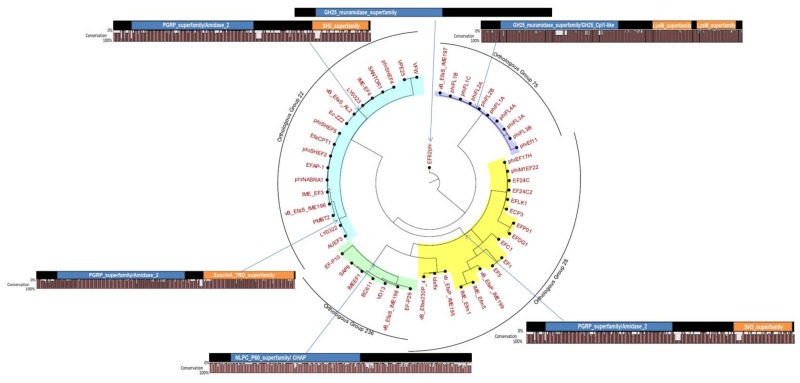
Maximum likelihood phylogenetic analysis sequence relatedness of *E. faecalis* phage endolysin functional domains; tree node labels represent the bootstrap values; the sequence similarity between functional domains is evidenced by using identical filling patterns; in blue—active domain; in orange—biding in domain; each of the four orthologous group is represented by a different color; Ef62phi could not be associated to any orthologous group.

Based on their muralytic activity, four types of phage endolysins have already been identified; type I (lysozymes) and type II (transglycosidases); both of which act on the glycosidic bond linking the amino sugars in the cell wall. Type III (amidases) and type IV (endopeptidases), both act on the amide and peptide bonds of the oligopeptide cross-linking stems [110]. Endolysins typically consist of an N-terminal catalytic domain targeting the peptidoglycan network, and a C-terminal cell wall binding domain (termed as carbohydrate binding domain, CBD), which initializes the binding for corresponding enzymatic action, against the specific substrate (Loessner, 2005). A comprehensive in silico analysis on endolysin classes revealed that most (more than 74%) of the *E. faecalis* phage endolysins have an LysM module as a part of their Cell Binding Domain (CBD), whereas the Enzyme Catalytic Domain (ECD) consists of a glycosidase hydrolase (GH) module GH25 (the predominant one 50–74%) and cysteine, and hsitidine-dependent amidohydrolase/peptidase (CHAP) (accounting for less than 25%) (Oliveira et al. [111]). We identified a total of 54 putative and reference endolysin sequences in *E. faecalis* phages (Figure 3). They were clustered into orthologous groups (OGs) using OrthoMCL with default settings (Li et al. [57]). All but one (an endolysin associated with the phage EF62phi) clustered into one of the four distinct orthologous groups (OG 22, OG 28, OG 78, and OG 236), which mirrored the orthologous groups of their parental phages (Figure 2 and Figure 3).

One representative sequence was selected from each OG and subjected to HHMER [112] or HHPRED [113] analysis, to determine the protein domain architecture. Proteins assigned to the same OG often displayed the identical domain architectures, although a few exceptions were observed. In the case of ECD, three major domains—GH25, Amidase_2, and CHAP—were observed across the four OGs, whereas in CBD, three domains—LysM, SH3, and PET-M23 (ZoocinA)—were identified (Figure 3). This observation was consistent with the findings of Oliveira et al. [114].

### 7.2. Applications of E. faecalis Phage Endolysins

Of note, endolysins could also be used in combination with traditional antibiotics to treat polyantibiotic-resistant bacterial pathogens. Many studies have shown the successful application of phage endolysins, in treating multi-drug resistant bacterial infections caused by *A. baumannii*, *S. aureus*, Methicillin resistance *S. aureus* (MRSA), *E. coli, Proteus mirabilis*, *Klebsiella*, *Pseudomonas*, *Morganella*, *Enterobacter*, *Enterococcus*, and *Salmonella* [111]. A small number of studies have demonstrated the in vivo efficacy of *E. faecalis* specific endolysins. One recent study evaluated endolysin LysEF-P10 to treat multi-drug resistant *E. faecalis* in a mouse model [92]. Here, a single intraperitoneal dose of 5 µg LysEF-P10 endolysin, was sufficient to eliminate the vancomycin resistant strain from the gut, without causing any collateral damage to the gut communities. Another study described the use of the endolysin IME-EF1, which protected 80% of mice challenged with a lethal dose of *E. faecalis* 002, and significantly reduced bacterial proliferation in the blood [52]. Several studies have described the in vitro antimicrobial action of *E. faecalis* endolysins. Heterologous expression of two endolysins Lys168 and Lys170 derived from *E. faecalis*, displayed a promising activity against clinical isolates of exponentially growing vancomycin-resistant and sensitive *E. faecalis* cultures, but failed to display a similar activity against log phase cultures [62]. Lys170 contains a catalytic domain of the amidase-2 family, which has an N-acetlymuramoyl-L-alanine amidase activity, while Lys168 was identified as being unique among the enterococcal phage endolysins, and highly similar to the endolysin of *S. aureus* phage SAP6, therefore, distantly related to all CHAP domain containing enterococcal endolysins [62]. In a follow-up study, these authors used a domain shuffling approach, by fusing a peptidase M23 catalytic domain to a cell-wall-binding domain of the native endolysin Lys170, to generate a bacteriolysin-like chimera, designated as EC300, to improve its anti- *E. faecalis* activity [115]. A recent study highlighted the advantage of using the phage endolysin IME-EFm5, over a narrow host range *E. faecalis* phage. Interestingly, the endolysin of phage IME-EFm5, displayed lytic activity against almost all tested strains [15]. Similarly, an expanded lytic activity of the *E. faecalis* bacteriophage ɸEF24C endolysin, ORF9 has been observed when heterologously expressed in *E. coli*. Further analysis has revealed that ORF9 belongs to the family of N-acetlymuramoyl-L-alanine amidases [44,116]. 

Antibacterial activity of a thermostable endolysin VD13 with an N-terminal CHAP domain has been demonstrated in vitro, against *E. faecalis*, with no activity observed against *E. faecium* or any other non-enterococcal strains tested [51]. In general, phage endolysins display a wider spectrum of activity than their parental phage counterparts. 

## 8. Conclusions

We conclude that phages could provide a viable alternative therapy to antibiotics in the fight against *E. faecalis* infections. To date, only one clinical study has demonstrated the efficiency of *E. faecalis* phages in a clinical setting. However, there are increased chances of developing a successful phage therapy approach to an *E. faecalis* control, based on the in vitro and in vivo studies described in this review. As far as we are aware, no current phage clinical trials are focused on *E. faecalis*, but the outcomes of trials targeting other pathogens might be useful for the design of future *E. faecalis* phage therapy. 

One of the issues of phage therapy is the narrow host range of the phages. In the case of *E. faecalis*, the diversity of phages showed in this review, based on the orthocluster identification, support the idea of expanding the phage host range by creating phage cocktails with a broader host range. It is unlikely that resistance will simultaneously occur for all virulent phages.

If this approach fails, there is the possibility of engineering temperate phages, as was done successfully for the *E. faecalis* phage phiEf11. Moreover, even if phages fail in providing a therapy for *E. faecalis*, their endolysins might prove to be a suitable alternative in the fight against *E. faecalis*-associated disease.

## Figures and Tables

**Table 1:** Target infections, phage dosage, and outcomes in *Enterococcus faecalis* phage therapy in vivo models.

Disease (Target Strain)	No (n) and Type of Subjects	Form and Dosage	Application Route and Clinical Outcome	Reference
Chronic bacterial prostatitis	*n* = 3 human male	Phage lysate ~10^7^–10^9^ PFU/mL	Rectal Pathogen eradication, Abatement of clinical symptoms Lack of early disease recurrence	[88]
Infection (EF14 VRE2)	*n* = 20; BALB/c mice female 6 to 8 week old	CsCl; 1 × 10^12^ PFU/mL;	Intraperitoneal; Significantly effective, Efficiently rescued mice;	[89]
Bacteremia (VAN)	*n* = 5 BALB/c mice 1 month old	CsCl 3 × 10^8^ PFU/mL	Intraperitoneal 100% survival 45 min after bacterial challenge 50% of moribund mice rescued after delayed phage administration	[90]
Sepsis 002	*n* = 8 7 different dosage groups BALB/c female mice 6 to 8 weeks old	PEG 3.9 × 10^9^ PFU/mL or 0.2 mg endolysin	Intraperitoneal 60% survival at 30 min post bacterial inoculation 40% survival at 4 h post bacterial administration	[52]
*E. faecalis* challenge	*n* = 10 5 different dosage groups BALB/c F 6 to 8 weeks old	CsCl 4 × 10^3^, 4 × 10^4^, 4 × 10^5^, 4 × 10^6^, 4 × 10^7^ PFU/mouse	Intraperitoneal Mice were protected from the infection	[91]
Septic peritonitis	*n* = 15 4 groups ICR(CD-1C)	Dialyzed phage lysate 2 × 10^8^	Intraperitoneal 100% survival No harmful effect on the microbiome	[60]
*E. faecalis* challenge (VAN)	*n* = 5 4 different groups BALB/c n female mice 6 to 8 weeks old	LysEF-P10 endolysin 1 µg, 5 µg, 10 µg	Intraperitoneal Reduced *E. faecalis* colonization Alleviated the gut microbiota imbalance caused by VRE	[92]

VAN- experiment performed using vancomycin resistant *E. faecalis*; CsCl- Cesium chloride gradient purified phages; PEG- phage prepared by PEG precipitation.

## Supplementary Materials

The following are available online at https://www.mdpi.com/1999-4915/11/4/366/s1. Table S1: *E. faecalis* phages selected for the OrthoMCL analyses.
