# Supplementary material for: Evaluation of Phage Therapy in the Context of Enterococcus faecalis and Its Associated Diseases
Source: Viruses. 2019 Apr 20;11(4):366. doi: 10.3390/v11040366 (PMC6521178; doi:10.3390/v11040366)
Supplement: Supplementary file 1 [file viruses-11-00366-s001.pdf]

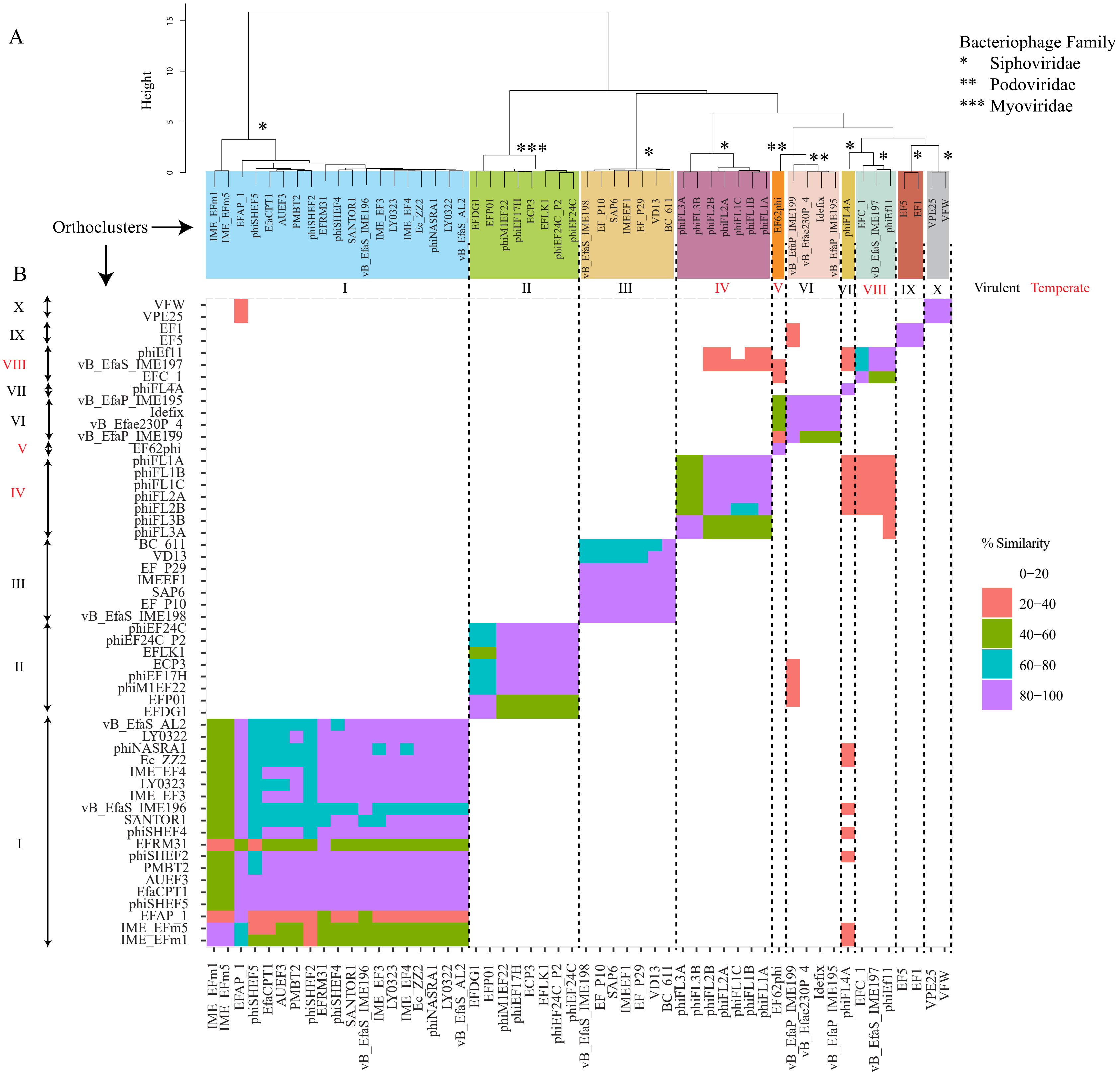

A
B
Height
Bacteriophage Family
* Siphoviridae
** Podoviridae
*** Myoviridae
Orthoclusters
I
II
III
IV
V
VI
VII
VIII
IX
X
Virulent
Temperate
% Similarity
0–20
20–40
40–60
60–80
80–100

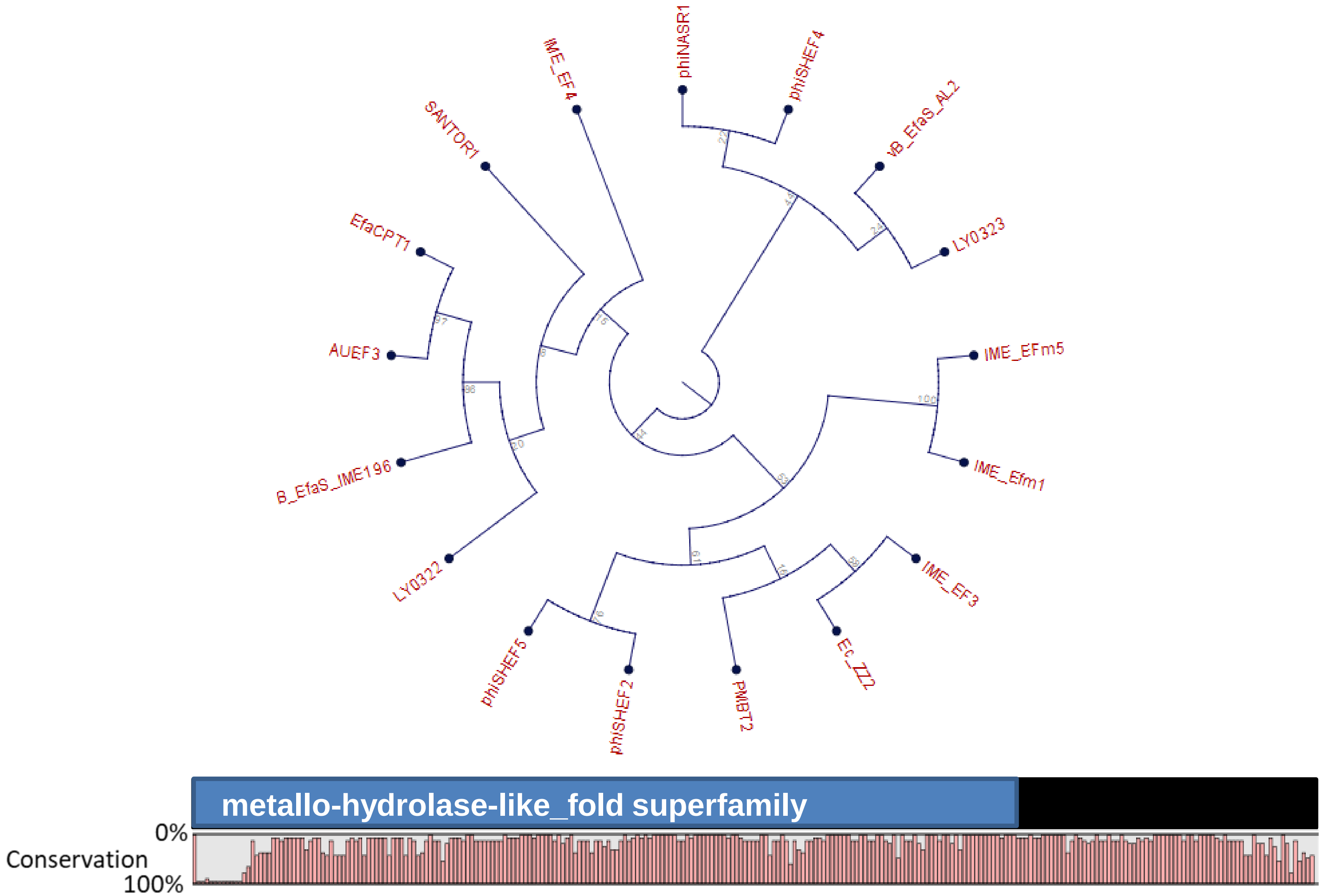
phiNASR1
phiSHEF4
vB_EfaS_AL2
LY0323
IME_EFm5
IME_Efm1
IME_EF3
Ec_ZZ2
PMBT2
phiSHEF2
phiSHEF5
LY0322
B_EfaS_IME196
AUEF3
EfaCPT1
SANTOR1
IME_EF4
metallo-hydrolase-like_fold superfamily
0%
Conservation
100%

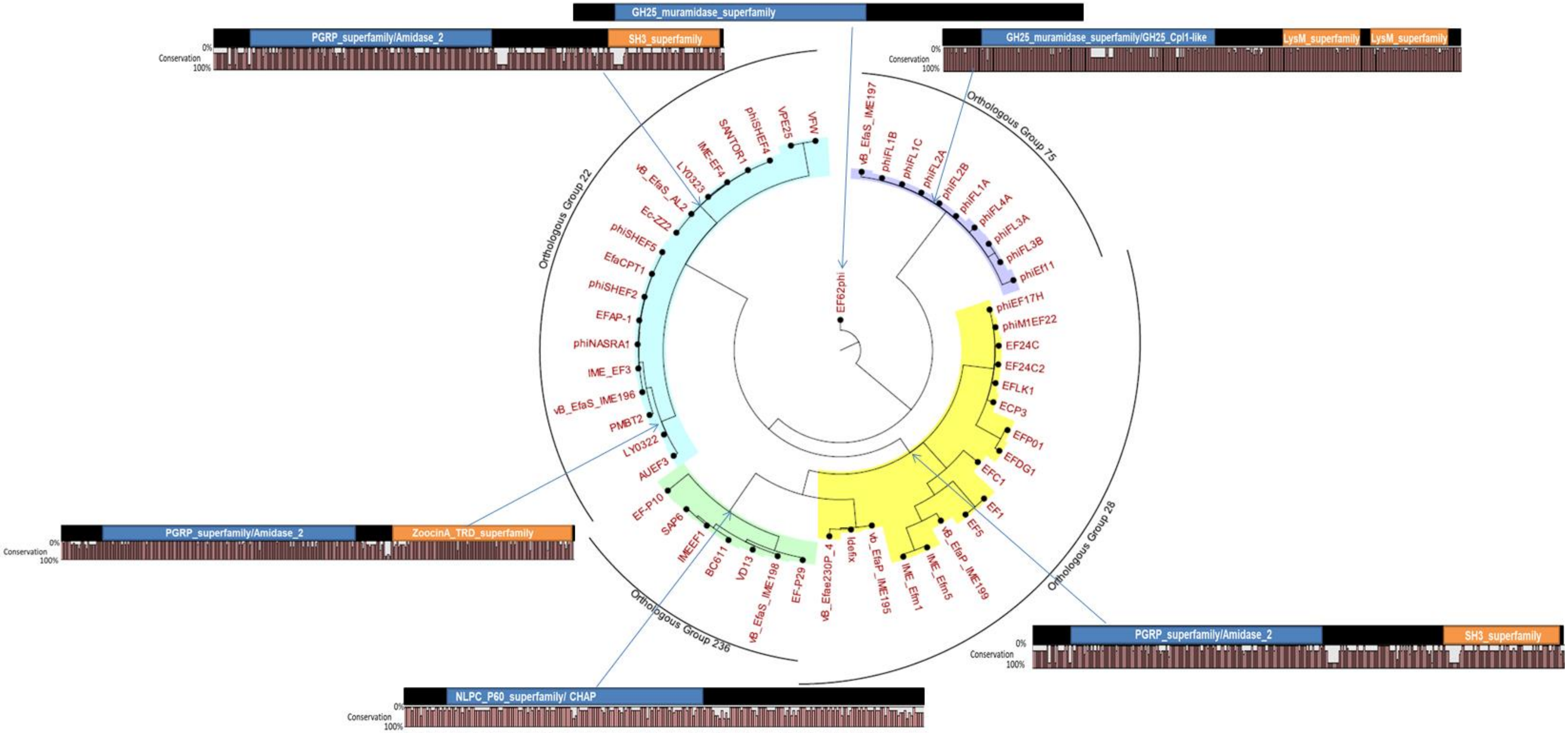
GH25_muramidase_superfamily
PGRP_superfamily/Amidase_2
SH3_superfamily
GH25_muramidase_superfamily/GH25_Cpl1-like
LysM_superfamily
LysM_superfamily
PGRP_superfamily/Amidase_2
ZoocinA_TRD_superfamily
PGRP_superfamily/Amidase_2
SH3_superfamily
NLPC_P60_superfamily/ CHAP
Conservation
0%
100%
Orthologous Group 22
Orthologous Group 75
Orthologous Group 28
Orthologous Group 236
EF62phi
vB_EfaS_IME197
phiFL1B
phiFL1C
phiFL2A
phiFL2B
phiFL1A
phiFL4A
phiFL3A
phiFL3B
phiEf11
phiEF17H
phiM1EF22
EF24C
EF24C2
EFLK1
ECP3
EFP01
EFDG1
EFC1
EF1
EF5
vB_EfaP_IME199
IME_Efm5
IME_Efm1
vb_EfaP_IME195
Idefix
vB_Efae230P_4
EF-P29
vB_EfaS_IME198
VD13
BC611
IMEEF1
SAP6
EF-P10
AUEF3
LY0322
PMBT2
vB_EfaS_IME196
IME_EF3
phiNASRA1
EFAP-1
phiSHEF2
EfaCPT1
phiSHEF5
Ec-ZZ2
vB_EfaS_AL2
LY0323
IME-EF4
SANTOR1
phiSHEF4
VPE25
VFW
